# Supplementary material for: The identification of dual protective agents against cisplatin-induced oto- and nephrotoxicity using the zebrafish model
Source: eLife. 2020 Jul 28;9:e56235. doi: 10.7554/eLife.56235 (PMC7470826; doi:10.7554/eLife.56235)
Supplement: Supplementary file 1. — Clinical usage information obtained from the ChEMBL database (Gaulton et al., 2017) [file elife-56235-supp1.docx]

**Supplementary File 1 Compounds from the Sigma LOPAC®^1280^ compound library that were toxic to ¾ zebrafish larvae at 0.01mM, in combination with 0.02mM cisplatin.** Clinical usage information obtained from the ChEMBL database (Gaulton et al., 2017)

| **Drug ID** | **Drug Name** | **Biological Action** | **Clinical Usage** | **Hit in *in vitro* nephrotox assay** |
| --- | --- | --- | --- | --- |
| 1, B03 | PD 0325901 | PD 0325901 is a potent MKK1 (MEK1) and MKK2 (MEK2) inhibitor | Investigated in a Phase II clinical trial in non-small cell lung cancer (NSCLC), did not meet efficacy endpoint (Haura et al., 2010). Investigated for potential utility in neurofibromatosis type 1 (NCT02096471) and colorectal cancer (NCT02510001). | Yes |
| 1, G02 | Tryptamine hydrochloride | Serotonin receptor ligand | N/A | Yes |
| 4, A07 | Cyproheptadine hydrochloride | 5-HT2 serotonin receptor antagonist | First generation antihistamine used clinically for allergies, especially hayfever |  |
| 4, A09 | Cantharidin | Protein phosphatase 2A inhibitor | In clinical trials to treat molluscum (NCT03377803), listed as an extremely hazardous agent in the USA because it can cause severe chemical burns |  |
| 4, B05 | Chlorothiazide | Carbonic anhydrase inhibitor, diuretic; antihypertensive | Used clinically as a diuretic and antihypertensive | Yes |
| 4, H08 | Calcimycin | Ca^2+^ ionophore used to potentiate responses to NMDA, but not quisqualate glutamate receptors | N/A |  |
| 4, H09 | Cantharidic Acid | Protein phosphatase 1 (PP1) and 2A (PP2A) inhibitor | N/A |  |
| 5, E11 | Diacylglycerol Kinase Inhibitor II | Diacylglycerol kinase inhibitor | N/A |  |
| 5, H03 | (+)-Chloro-APB hydrobromide | D1 dopamine receptor antagonist | N/A |  |
| 5, H10 | 2,3-Dimethoxy-1,4-naphthoquinone | Redox cycling agent used to study role of ROS | N/A |  |
| 6, A08 | 4-DAMP methiodide | M3 muscarinic acetylcholine receptor antagonist | N/A | Yes |
| 6, D11 | Etazolate hydrochloride | Phosphodiesterase inhibitor | Tested in Phase II clinical trial for adjunct therapy in Alzheimer’s disease (NCT00880412), suggested to have anxiolytic effects | Yes |
| 6, F03 | DBO-83 | Nicotinic acetylcholine receptor agonist | N/A |  |
| 6, H06 | AC-93253 iodide | Potent, cell permeable, subtype selective retinoic acid receptor (RARalpha) agonist | Studied experimentally *in vitro* and in mice for the treatment of NSCLC (Lai, Lin, Wu, Chen, & Chen, 2017) | Yes |
| 7, F11 | NS5806 | Increases peak current amplitude of the potassium channel Kv4.3 |  | Yes |
| 7, H09 | Fluoxetine hydrochloride | Selective serotonin reuptake inhibitor | Used for the treatment of major depressive disorder, panic disorder, obsessive-compulsive disorder etc. (aka. Prozac) |  |
| 10, B06 | BBMP | Mitochondrial permeability transition pore (PTP) inhibitor. Potential therapeutics for neurodegenerative diseases | N/A |  |
| 16, E09 | Wortmannin from Penicillum funiculosum | Potent and specific phosphatidylinositol 3-kinase (PI3-K) inhibitor | N/A |  |
| 16, F05 | Tyrphostin A9 | Selective PDGF tyrosine kinase receptor inhibitor | N/A |  |
