# Supplementary material for: The identification of dual protective agents against cisplatin-induced oto- and nephrotoxicity using the zebrafish model
Source: eLife. 2020 Jul 28;9:e56235. doi: 10.7554/eLife.56235 (PMC7470826; doi:10.7554/eLife.56235)
Supplement: Supplementary file 2. [file elife-56235-supp2.docx]

|  | **Figure 3a** |  |  |  |  |  |  |
| --- | --- | --- | --- | --- | --- | --- | --- |
|  | **Dunn's multiple comparisons test** | **Mean rank diff.** | **Significant?** | **Summary** | **Adjusted P Value** |  |  |
|  | Control vs. Cisplatin (0.01mM) | 376.2 | Yes | **** | <0.0001 |  |  |
|  | Control vs. Cisplatin (0.02mM) | 758.2 | Yes | **** | <0.0001 |  |  |
|  | Control vs. Cisplatin (0.02mM) + Dopamine (0.03mM) | 640.8 | Yes | **** | <0.0001 |  |  |
|  | Control vs. Cisplatin (0.02mM) + L-Mimosine (0.03mM) | 593.9 | Yes | **** | <0.0001 |  |  |
|  | Control vs. Cisplatin (0.02mM) + L-Mimosine (0.04mM) | 390 | Yes | **** | <0.0001 |  |  |
|  | Control vs. Cisplatin (0.02mM) + Dopamine (0.02mM) + L-Mimosine (0.02mM) | 470.3 | Yes | **** | <0.0001 |  |  |
|  | Cisplatin (0.01mM) vs. Cisplatin (0.02mM) | 382 | Yes | **** | <0.0001 |  |  |
|  | Cisplatin (0.01mM) vs. Cisplatin (0.02mM) + Dopamine (0.03mM) | 264.6 | Yes | **** | <0.0001 |  |  |
|  | Cisplatin (0.01mM) vs. Cisplatin (0.02mM) + L-Mimosine (0.03mM) | 217.7 | Yes | **** | <0.0001 |  |  |
|  | Cisplatin (0.01mM) vs. Cisplatin (0.02mM) + L-Mimosine (0.04mM) | 13.83 | No | ns | >0.9999 |  |  |
|  | Cisplatin (0.01mM) vs. Cisplatin (0.02mM) + Dopamine (0.02mM) + L-Mimosine (0.02mM) | 94.12 | No | ns | 0.5678 |  |  |
|  | Cisplatin (0.02mM) vs. Cisplatin (0.02mM) + Dopamine (0.03mM) | -117.4 | Yes | * | 0.0129 |  |  |
|  | Cisplatin (0.02mM) vs. Cisplatin (0.02mM) + L-Mimosine (0.03mM) | -164.3 | Yes | **** | <0.0001 |  |  |
|  | Cisplatin (0.02mM) vs. Cisplatin (0.02mM) + L-Mimosine (0.04mM) | -368.2 | Yes | **** | <0.0001 |  |  |
|  | Cisplatin (0.02mM) vs. Cisplatin (0.02mM) + Dopamine (0.02mM) + L-Mimosine (0.02mM) | -287.9 | Yes | **** | <0.0001 |  |  |
|  | Cisplatin (0.02mM) + Dopamine (0.03mM) vs. Cisplatin (0.02mM) + L-Mimosine (0.03mM) | -46.94 | No | ns | >0.9999 |  |  |
|  | Cisplatin (0.02mM) + Dopamine (0.03mM) vs. Cisplatin (0.02mM) + L-Mimosine (0.04mM) | -250.8 | Yes | **** | <0.0001 |  |  |
|  | Cisplatin (0.02mM) + Dopamine (0.03mM) vs. Cisplatin (0.02mM) + Dopamine (0.02mM) + L-Mimosine (0.02mM) | -170.5 | Yes | **** | <0.0001 |  |  |
|  | Cisplatin (0.02mM) + L-Mimosine (0.03mM) vs. Cisplatin (0.02mM) + L-Mimosine (0.04mM) | -203.9 | Yes | **** | <0.0001 |  |  |
|  | Cisplatin (0.02mM) + L-Mimosine (0.03mM) vs. Cisplatin (0.02mM) + Dopamine (0.02mM) + L-Mimosine (0.02mM) | -123.6 | Yes | ** | 0.0056 |  |  |
|  | Cisplatin (0.02mM) + L-Mimosine (0.04mM) vs. Cisplatin (0.02mM) + Dopamine (0.02mM) + L-Mimosine (0.02mM) | 80.29 | No | ns | 0.3834 |  |  |
|  |  |  |  |  |  |  |  |
|  |  |  |  |  |  |  |  |
|  | **Figure 4e** |  |  |  |  |  |  |
|  | **Dunn's multiple comparisons test** | **Mean rank diff.** | **Significant?** | **Summary** | **Adjusted P Value** |  |  |
|  | Control vs. Cisplatin | 93.69 | Yes | **** | <0.0001 |  |  |
|  | Control vs. Cisplatin + LDOPA | 24.47 | No | ns | 0.7601 |  |  |
|  | Control vs. Cisplatin + MIM | 40.93 | No | ns | 0.0638 |  |  |
|  | Cisplatin vs. Cisplatin + LDOPA | -69.22 | Yes | **** | <0.0001 |  |  |
|  | Cisplatin vs. Cisplatin + MIM | -52.76 | Yes | ** | 0.0059 |  |  |
|  | Cisplatin + LDOPA vs. Cisplatin + MIM | 16.46 | No | ns | >0.9999 |  |  |
|  |  |  |  |  |  |  |  |
|  | **Figure 4f** |  |  |  |  |  |  |
|  | **Tukey's multiple comparisons test** | **Mean Diff.** | **95.00% CI of diff.** | **Significant?** | **Summary** | **Adjusted P Value** | |
|  | Control vs. Cisplatin | -0.1181 | -0.1600 to -0.07621 | Yes | **** | <0.0001 |  |
|  | Control vs. Cisplatin + LDOPA | -0.04438 | -0.08627 to -0.002486 | Yes | * | 0.0331 |  |
|  | Control vs. Cisplatin + MIM | -0.03896 | -0.08085 to 0.002931 | No | ns | 0.0788 |  |
|  | Cisplatin vs. Cisplatin + LDOPA | 0.07372 | 0.03183 to 0.1156 | Yes | **** | <0.0001 |  |
|  | Cisplatin vs. Cisplatin + MIM | 0.07914 | 0.03725 to 0.1210 | Yes | **** | <0.0001 |  |
|  | Cisplatin + LDOPA vs. Cisplatin + MIM | 0.005417 | -0.03647 to 0.04731 | No | ns | 0.9872 |  |
|  |  |  |  |  |  |  |  |
|  |  |  |  |  |  |  |  |
|  | **Figure 5a** |  |  |  |  |  |  |
|  | **Tukey's multiple comparisons test** | **Predicted (LS) mean diff.** | **95.00% CI of diff.** | **Significant?** | **Summary** | **Adjusted P Value** | |
|  |  |  |  |  |  |  |  |
|  | 0:Control vs. 0:0.125mM Cisplatin Alone | 0.02918 | -0.01975 to 0.07811 | No | ns | 0.6134 |  |
|  | 0:Control vs. 0:0.125mM Cisplatin + 0.03mM Dopamine | 0.02418 | -0.02083 to 0.06919 | No | ns | 0.7316 |  |
|  | 0:Control vs. 0:0.125 mMCisplatin + 0.03mM L-Mimosine | 0.02331 | -0.02271 to 0.06933 | No | ns | 0.787 |  |
|  | 0:Control vs. 2:Control | 0.1458 | 0.09952 to 0.1921 | Yes | **** | <0.0001 |  |
|  | 0:Control vs. 2:0.125mM Cisplatin Alone | 0.06964 | 0.01951 to 0.1198 | Yes | *** | 0.0007 |  |
|  | 0:Control vs. 2:0.125mM Cisplatin + 0.03mM Dopamine | 0.1026 | 0.05646 to 0.1487 | Yes | **** | <0.0001 |  |
|  | 0:Control vs. 2:0.125 mMCisplatin + 0.03mM L-Mimosine | 0.11 | 0.06345 to 0.1565 | Yes | **** | <0.0001 |  |
|  | 0:0.125mM Cisplatin Alone vs. 0:0.125mM Cisplatin + 0.03mM Dopamine | -0.004998 | -0.05432 to 0.04432 | No | ns | >0.9999 |  |
|  | 0:0.125mM Cisplatin Alone vs. 0:0.125 mMCisplatin + 0.03mM L-Mimosine | -0.00587 | -0.05611 to 0.04437 | No | ns | >0.9999 |  |
|  | 0:0.125mM Cisplatin Alone vs. 2:Control | 0.1166 | 0.06614 to 0.1671 | Yes | **** | <0.0001 |  |
|  | 0:0.125mM Cisplatin Alone vs. 2:0.125mM Cisplatin Alone | 0.04046 | -0.01357 to 0.09450 | No | ns | 0.3093 |  |
|  | 0:0.125mM Cisplatin Alone vs. 2:0.125mM Cisplatin + 0.03mM Dopamine | 0.07342 | 0.02307 to 0.1238 | Yes | *** | 0.0003 |  |
|  | 0:0.125mM Cisplatin Alone vs. 2:0.125 mMCisplatin + 0.03mM L-Mimosine | 0.08082 | 0.03009 to 0.1315 | Yes | **** | <0.0001 |  |
|  | 0:0.125mM Cisplatin + 0.03mM Dopamine vs. 0:0.125 mMCisplatin + 0.03mM L-Mimosine | -0.0008721 | -0.04730 to 0.04555 | No | ns | >0.9999 |  |
|  | 0:0.125mM Cisplatin + 0.03mM Dopamine vs. 2:Control | 0.1216 | 0.07493 to 0.1683 | Yes | **** | <0.0001 |  |
|  | 0:0.125mM Cisplatin + 0.03mM Dopaminevs. 2:0.125mM Cisplatin Alone | 0.04546 | -0.005046 to 0.09597 | No | ns | 0.1138 |  |
|  | 0:0.125mM Cisplatin + 0.03mM Dopamine vs. 2:0.125mM Cisplatin + 0.03mM Dopamine | 0.07842 | 0.03187 to 0.1250 | Yes | **** | <0.0001 |  |
|  | 0:0.125mM Cisplatin + 0.03mM Dopaminevs. 2:0.125 mMCisplatin + 0.03mM L-Mimosine | 0.08581 | 0.03886 to 0.1328 | Yes | **** | <0.0001 |  |
|  | 0:0.125 mMCisplatin + 0.03mM L-Mimosine vs. 2:Control | 0.1225 | 0.07483 to 0.1701 | Yes | **** | <0.0001 |  |
|  | 0:0.125 mMCisplatin + 0.03mM L-Mimosine vs. 2:0.125mM Cisplatin Alone | 0.04633 | -0.005070 to 0.09774 | No | ns | 0.1128 |  |
|  | 0:0.125 mMCisplatin + 0.03mM L-Mimosine vs. 2:0.125mM Cisplatin + 0.03mM Dopamine | 0.07929 | 0.03177 to 0.1268 | Yes | **** | <0.0001 |  |
|  | 0:0.125 mMCisplatin + 0.03mM L-Mimosinevs. 2:0.125 mMCisplatin + 0.03mM L-Mimosine | 0.08669 | 0.03877 to 0.1346 | Yes | **** | <0.0001 |  |
|  | 2:Control vs. 2:0.125mM Cisplatin Alone | -0.07615 | -0.1278 to -0.02451 | Yes | *** | 0.0002 |  |
|  | 2:Control vs. 2:0.125mM Cisplatin + 0.03mM Dopamine | -0.04319 | -0.09097 to 0.004584 | No | ns | 0.1105 |  |
|  | 2:Control vs. 2:0.125 mMCisplatin + 0.03mM L-Mimosine | -0.0358 | -0.08396 to 0.01236 | No | ns | 0.3189 |  |
|  | 2:0.125mM Cisplatin Alone vs. 2:0.125mM Cisplatin + 0.03mM Dopamine | 0.03296 | -0.01856 to 0.08448 | No | ns | 0.5221 |  |
|  | 2:0.125mM Cisplatin Alone vs. 2:0.125 mMCisplatin + 0.03mM L-Mimosine | 0.04035 | -0.01153 to 0.09223 | No | ns | 0.2616 |  |
|  | 2:0.125mM Cisplatin + 0.03mM Dopamine vs. 2:0.125 mMCisplatin + 0.03mM L-Mimosine | 0.007392 | -0.04064 to 0.05543 | No | ns | 0.9998 |  |
|  | **Figure 5a - Multiple comparisons test performed to double check** |  |  |  |  |  |  |
|  | **Sidak's multiple comparisons test** | **Predicted (LS) mean diff.** | **95.00% CI of diff.** | **Significant?** | **Summary** | **Adjusted P Value** | |
|  | 0 - 2 |  |  |  |  |  |  |
|  | Control | 0.1458 | 0.1078 to 0.1838 | Yes | **** | <0.0001 |  |
|  | 0.125mM Cisplatin Alone | 0.04046 | -0.003934 to 0.08486 | No | ns | 0.0895 |  |
|  | 0.125mM Cisplatin + 0.03mM Dopamine | 0.07842 | 0.04017 to 0.1167 | Yes | **** | <0.0001 |  |
|  | 0.125 mMCisplatin + 0.03mM L-Mimosine | 0.08669 | 0.04732 to 0.1261 | Yes | **** | <0.0001 |  |
|  |  |  |  |  |  |  |  |
|  |  |  |  |  |  |  |  |
|  | **Figure 6a** |  |  |  |  |  |  |
| LAN5 Dopamine 48h | **Tukey's multiple comparisons test** | Mean Diff. | 95.00% CI of diff. | Significant? | Summary | Adjusted P Value |  |
|  |  |  |  |  |  |  |  |
|  | Row 1 |  |  |  |  |  |  |
|  | 0mM Dopamine vs. 0.01mM Dopamine | 15.26 | -2.454 to 32.97 | No | ns | 0.104 |  |
|  | 0mM Dopamine vs. 0.03mM Dopamine | 85.37 | 67.66 to 103.1 | Yes | **** | <0.0001 |  |
|  | 0.01mM Dopamine vs. 0.03mM Dopamine | 70.11 | 52.40 to 87.82 | Yes | **** | <0.0001 |  |
|  |  |  |  |  |  |  |  |
|  | Row 2 |  |  |  |  |  |  |
|  | 0mM Dopamine vs. 0.01mM Dopamine | 15.9 | -1.813 to 33.61 | No | ns | 0.0866 |  |
|  | 0mM Dopamine vs. 0.03mM Dopamine | 83.83 | 66.11 to 101.5 | Yes | **** | <0.0001 |  |
|  | 0.01mM Dopamine vs. 0.03mM Dopamine | 67.93 | 50.22 to 85.64 | Yes | **** | <0.0001 |  |
|  |  |  |  |  |  |  |  |
|  | Row 3 |  |  |  |  |  |  |
|  | 0mM Dopamine vs. 0.01mM Dopamine | 8.729 | -8.983 to 26.44 | No | ns | 0.4637 |  |
|  | 0mM Dopamine vs. 0.03mM Dopamine | 65.24 | 47.53 to 82.95 | Yes | **** | <0.0001 |  |
|  | 0.01mM Dopamine vs. 0.03mM Dopamine | 56.51 | 38.80 to 74.22 | Yes | **** | <0.0001 |  |
|  |  |  |  |  |  |  |  |
|  | Row 4 |  |  |  |  |  |  |
|  | 0mM Dopamine vs. 0.01mM Dopamine | 1.697 | -16.01 to 19.41 | No | ns | 0.9709 |  |
|  | 0mM Dopamine vs. 0.03mM Dopamine | 32.82 | 15.11 to 50.53 | Yes | *** | 0.0001 |  |
|  | 0.01mM Dopamine vs. 0.03mM Dopamine | 31.12 | 13.41 to 48.83 | Yes | *** | 0.0003 |  |
|  |  |  |  |  |  |  |  |
|  | Row 5 |  |  |  |  |  |  |
|  | 0mM Dopamine vs. 0.01mM Dopamine | 6.829 | -10.88 to 24.54 | No | ns | 0.6226 |  |
|  | 0mM Dopamine vs. 0.03mM Dopamine | 23.9 | 6.186 to 41.61 | Yes | ** | 0.0057 |  |
|  | 0.01mM Dopamine vs. 0.03mM Dopamine | 17.07 | -0.6428 to 34.78 | No | ns | 0.0611 |  |
|  |  |  |  |  |  |  |  |
|  | Row 6 |  |  |  |  |  |  |
|  | 0mM Dopamine vs. 0.01mM Dopamine | -1.917 | -19.63 to 15.79 | No | ns | 0.9629 |  |
|  | 0mM Dopamine vs. 0.03mM Dopamine | 8.308 | -9.403 to 26.02 | No | ns | 0.4979 |  |
|  | 0.01mM Dopamine vs. 0.03mM Dopamine | 10.23 | -7.486 to 27.94 | No | ns | 0.3509 |  |
|  |  |  |  |  |  |  |  |
|  | Row 7 |  |  |  |  |  |  |
|  | 0mM Dopamine vs. 0.01mM Dopamine | 0.4341 | -17.28 to 18.15 | No | ns | 0.9981 |  |
|  | 0mM Dopamine vs. 0.03mM Dopamine | 2.956 | -14.76 to 20.67 | No | ns | 0.9143 |  |
|  | 0.01mM Dopamine vs. 0.03mM Dopamine | 2.522 | -15.19 to 20.23 | No | ns | 0.9368 |  |
|  |  |  |  |  |  |  |  |
|  | Row 8 |  |  |  |  |  |  |
|  | 0mM Dopamine vs. 0.01mM Dopamine | 0.19 | -17.52 to 17.90 | No | ns | 0.9996 |  |
|  | 0mM Dopamine vs. 0.03mM Dopamine | 2.172 | -15.54 to 19.88 | No | ns | 0.9527 |  |
|  | 0.01mM Dopamine vs. 0.03mM Dopamine | 1.982 | -15.73 to 19.69 | No | ns | 0.9605 |  |
|  |  |  |  |  |  |  |  |
|  | **Figure 6b** |  |  |  |  |  |  |
| SK-N-AS Dopamine 48h | **Tukey's multiple comparisons test** | Predicted (LS) mean diff. | 95.00% CI of diff. | Significant? | Summary | Adjusted P Value |  |
|  |  |  |  |  |  |  |  |
|  | Row 1 |  |  |  |  |  |  |
|  | 0mM Dopamine vs. 0.01mM Dopamine | 15.9 | -15.11 to 46.91 | No | ns | 0.4378 |  |
|  | 0mM Dopamine vs. 0.03mM Dopamine | 47.44 | 14.29 to 80.58 | Yes | ** | 0.0031 |  |
|  | 0.01mM Dopamine vs. 0.03mM Dopamine | 31.54 | 0.5295 to 62.54 | Yes | * | 0.0454 |  |
|  |  |  |  |  |  |  |  |
|  | Row 2 |  |  |  |  |  |  |
|  | 0mM Dopamine vs. 0.01mM Dopamine | 1.267 | -29.74 to 32.27 | No | ns | 0.9947 |  |
|  | 0mM Dopamine vs. 0.03mM Dopamine | 13.19 | -19.95 to 46.34 | No | ns | 0.6058 |  |
|  | 0.01mM Dopamine vs. 0.03mM Dopamine | 11.93 | -19.08 to 42.93 | No | ns | 0.626 |  |
|  |  |  |  |  |  |  |  |
|  | Row 3 |  |  |  |  |  |  |
|  | 0mM Dopamine vs. 0.01mM Dopamine | 8.98 | -22.03 to 39.99 | No | ns | 0.7659 |  |
|  | 0mM Dopamine vs. 0.03mM Dopamine | 25.83 | -7.320 to 58.97 | No | ns | 0.1551 |  |
|  | 0.01mM Dopamine vs. 0.03mM Dopamine | 16.85 | -14.16 to 47.85 | No | ns | 0.3964 |  |
|  |  |  |  |  |  |  |  |
|  | Row 4 |  |  |  |  |  |  |
|  | 0mM Dopamine vs. 0.01mM Dopamine | 3.505 | -27.50 to 34.51 | No | ns | 0.96 |  |
|  | 0mM Dopamine vs. 0.03mM Dopamine | 17.35 | -15.80 to 50.50 | No | ns | 0.4233 |  |
|  | 0.01mM Dopamine vs. 0.03mM Dopamine | 13.84 | -17.16 to 44.85 | No | ns | 0.5332 |  |
|  |  |  |  |  |  |  |  |
|  | Row 5 |  |  |  |  |  |  |
|  | 0mM Dopamine vs. 0.01mM Dopamine | 6.594 | -24.41 to 37.60 | No | ns | 0.8657 |  |
|  | 0mM Dopamine vs. 0.03mM Dopamine | 25.37 | -7.780 to 58.51 | No | ns | 0.1652 |  |
|  | 0.01mM Dopamine vs. 0.03mM Dopamine | 18.77 | -12.23 to 49.78 | No | ns | 0.3187 |  |
|  |  |  |  |  |  |  |  |
|  | Row 6 |  |  |  |  |  |  |
|  | 0mM Dopamine vs. 0.01mM Dopamine | 7.375 | -27.78 to 42.53 | No | ns | 0.8691 |  |
|  | 0mM Dopamine vs. 0.03mM Dopamine | 25.11 | -11.95 to 62.17 | No | ns | 0.2409 |  |
|  | 0.01mM Dopamine vs. 0.03mM Dopamine | 17.74 | -13.27 to 48.74 | No | ns | 0.3593 |  |
|  |  |  |  |  |  |  |  |
|  | Row 7 |  |  |  |  |  |  |
|  | 0mM Dopamine vs. 0.01mM Dopamine | -2.49 | -33.50 to 28.52 | No | ns | 0.9796 |  |
|  | 0mM Dopamine vs. 0.03mM Dopamine | 23.21 | -9.939 to 56.36 | No | ns | 0.2194 |  |
|  | 0.01mM Dopamine vs. 0.03mM Dopamine | 25.7 | -5.307 to 56.71 | No | ns | 0.1226 |  |
|  |  |  |  |  |  |  |  |
|  | Row 8 |  |  |  |  |  |  |
|  | 0mM Dopamine vs. 0.01mM Dopamine | -1.644 | -32.65 to 29.36 | No | ns | 0.991 |  |
|  | 0mM Dopamine vs. 0.03mM Dopamine | 17.23 | -15.92 to 50.38 | No | ns | 0.4281 |  |
|  | 0.01mM Dopamine vs. 0.03mM Dopamine | 18.88 | -12.13 to 49.88 | No | ns | 0.3148 |  |
|  |  |  |  |  |  |  |  |
| HSC-3 Dopamine 48h | **Figure 6c** |  |  |  |  |  |  |
|  | **Tukey's multiple comparisons test** | Mean Diff. | 95.00% CI of diff. | Significant? | Summary | Adjusted P Value |  |
|  |  |  |  |  |  |  |  |
|  | Row 1 |  |  |  |  |  |  |
|  | 0mM Dopamine vs. 0.01mM Dopamine | 4.383 | -26.02 to 34.79 | No | ns | 0.9353 |  |
|  | 0mM Dopamine vs. 0.03mM Dopamine | 6.197 | -24.21 to 36.60 | No | ns | 0.875 |  |
|  | 0.01mM Dopamine vs. 0.03mM Dopamine | 1.814 | -28.59 to 32.22 | No | ns | 0.9886 |  |
|  |  |  |  |  |  |  |  |
|  | Row 2 |  |  |  |  |  |  |
|  | 0mM Dopamine vs. 0.01mM Dopamine | 7.909 | -22.50 to 38.32 | No | ns | 0.8049 |  |
|  | 0mM Dopamine vs. 0.03mM Dopamine | 9.458 | -20.95 to 39.86 | No | ns | 0.7337 |  |
|  | 0.01mM Dopamine vs. 0.03mM Dopamine | 1.549 | -28.86 to 31.96 | No | ns | 0.9917 |  |
|  |  |  |  |  |  |  |  |
|  | Row 3 |  |  |  |  |  |  |
|  | 0mM Dopamine vs. 0.01mM Dopamine | 9.703 | -20.70 to 40.11 | No | ns | 0.722 |  |
|  | 0mM Dopamine vs. 0.03mM Dopamine | 10.35 | -20.05 to 40.76 | No | ns | 0.6904 |  |
|  | 0.01mM Dopamine vs. 0.03mM Dopamine | 0.6505 | -29.76 to 31.06 | No | ns | 0.9985 |  |
|  |  |  |  |  |  |  |  |
|  | Row 4 |  |  |  |  |  |  |
|  | 0mM Dopamine vs. 0.01mM Dopamine | -12.16 | -42.57 to 18.24 | No | ns | 0.6007 |  |
|  | 0mM Dopamine vs. 0.03mM Dopamine | 3.35 | -27.06 to 33.76 | No | ns | 0.9617 |  |
|  | 0.01mM Dopamine vs. 0.03mM Dopamine | 15.51 | -14.89 to 45.92 | No | ns | 0.4393 |  |
|  |  |  |  |  |  |  |  |
|  | Row 5 |  |  |  |  |  |  |
|  | 0mM Dopamine vs. 0.01mM Dopamine | 8.278 | -22.13 to 38.68 | No | ns | 0.7885 |  |
|  | 0mM Dopamine vs. 0.03mM Dopamine | 18.05 | -12.35 to 48.46 | No | ns | 0.3309 |  |
|  | 0.01mM Dopamine vs. 0.03mM Dopamine | 9.774 | -20.63 to 40.18 | No | ns | 0.7186 |  |
|  |  |  |  |  |  |  |  |
|  | Row 6 |  |  |  |  |  |  |
|  | 0mM Dopamine vs. 0.01mM Dopamine | -5.775 | -36.18 to 24.63 | No | ns | 0.8905 |  |
|  | 0mM Dopamine vs. 0.03mM Dopamine | -3.035 | -33.44 to 27.37 | No | ns | 0.9684 |  |
|  | 0.01mM Dopamine vs. 0.03mM Dopamine | 2.74 | -27.67 to 33.15 | No | ns | 0.9742 |  |
|  |  |  |  |  |  |  |  |
|  | Row 7 |  |  |  |  |  |  |
|  | 0mM Dopamine vs. 0.01mM Dopamine | -1.132 | -31.54 to 29.27 | No | ns | 0.9955 |  |
|  | 0mM Dopamine vs. 0.03mM Dopamine | 6.322 | -24.08 to 36.73 | No | ns | 0.8703 |  |
|  | 0.01mM Dopamine vs. 0.03mM Dopamine | 7.454 | -22.95 to 37.86 | No | ns | 0.8246 |  |
|  |  |  |  |  |  |  |  |
|  | Row 8 |  |  |  |  |  |  |
|  | 0mM Dopamine vs. 0.01mM Dopamine | 1.362 | -29.04 to 31.77 | No | ns | 0.9936 |  |
|  | 0mM Dopamine vs. 0.03mM Dopamine | 3.532 | -26.87 to 33.94 | No | ns | 0.9575 |  |
|  | 0.01mM Dopamine vs. 0.03mM Dopamine | 2.17 | -28.24 to 32.58 | No | ns | 0.9837 |  |
|  |  |  |  |  |  |  |  |
| LAN5 L-Mimosine 48h | **Figure 6d** |  |  |  |  |  |  |
|  | **Tukey's multiple comparisons test** | Mean Diff. | 95.00% CI of diff. | Significant? | Summary | Adjusted P Value |  |
|  |  |  |  |  |  |  |  |
|  | Row 1 |  |  |  |  |  |  |
|  | 0mM L-Mimosine vs. 0.01mM L-Mimosine | 3.487 | -17.32 to 24.30 | No | ns | 0.9136 |  |
|  | 0mM L-Mimosine vs. 0.03mM L-Mimosine | 10.58 | -10.23 to 31.38 | No | ns | 0.442 |  |
|  | 0.01mM L-Mimosine vs. 0.03mM L-Mimosine | 7.089 | -13.72 to 27.90 | No | ns | 0.6901 |  |
|  |  |  |  |  |  |  |  |
|  | Row 2 |  |  |  |  |  |  |
|  | 0mM L-Mimosine vs. 0.01mM L-Mimosine | 8.434 | -12.37 to 29.24 | No | ns | 0.5927 |  |
|  | 0mM L-Mimosine vs. 0.03mM L-Mimosine | 16.46 | -4.349 to 37.27 | No | ns | 0.146 |  |
|  | 0.01mM L-Mimosine vs. 0.03mM L-Mimosine | 8.025 | -12.78 to 28.83 | No | ns | 0.6225 |  |
|  |  |  |  |  |  |  |  |
|  | Row 3 |  |  |  |  |  |  |
|  | 0mM L-Mimosine vs. 0.01mM L-Mimosine | 1.03 | -19.78 to 21.84 | No | ns | 0.9921 |  |
|  | 0mM L-Mimosine vs. 0.03mM L-Mimosine | 7.355 | -13.45 to 28.16 | No | ns | 0.671 |  |
|  | 0.01mM L-Mimosine vs. 0.03mM L-Mimosine | 6.325 | -14.48 to 27.13 | No | ns | 0.744 |  |
|  |  |  |  |  |  |  |  |
|  | Row 4 |  |  |  |  |  |  |
|  | 0mM L-Mimosine vs. 0.01mM L-Mimosine | 1.428 | -19.38 to 22.24 | No | ns | 0.9849 |  |
|  | 0mM L-Mimosine vs. 0.03mM L-Mimosine | 6.246 | -14.56 to 27.05 | No | ns | 0.7494 |  |
|  | 0.01mM L-Mimosine vs. 0.03mM L-Mimosine | 4.819 | -15.99 to 25.63 | No | ns | 0.8418 |  |
|  |  |  |  |  |  |  |  |
|  | Row 5 |  |  |  |  |  |  |
|  | 0mM L-Mimosine vs. 0.01mM L-Mimosine | -0.3135 | -21.12 to 20.49 | No | ns | 0.9993 |  |
|  | 0mM L-Mimosine vs. 0.03mM L-Mimosine | 0.1838 | -20.62 to 20.99 | No | ns | 0.9997 |  |
|  | 0.01mM L-Mimosine vs. 0.03mM L-Mimosine | 0.4973 | -20.31 to 21.31 | No | ns | 0.9982 |  |
|  |  |  |  |  |  |  |  |
|  | Row 6 |  |  |  |  |  |  |
|  | 0mM L-Mimosine vs. 0.01mM L-Mimosine | 0.1245 | -20.68 to 20.93 | No | ns | 0.9999 |  |
|  | 0mM L-Mimosine vs. 0.03mM L-Mimosine | 2.982 | -17.83 to 23.79 | No | ns | 0.936 |  |
|  | 0.01mM L-Mimosine vs. 0.03mM L-Mimosine | 2.857 | -17.95 to 23.67 | No | ns | 0.9411 |  |
|  |  |  |  |  |  |  |  |
|  | Row 7 |  |  |  |  |  |  |
|  | 0mM L-Mimosine vs. 0.01mM L-Mimosine | 0.5156 | -20.29 to 21.32 | No | ns | 0.998 |  |
|  | 0mM L-Mimosine vs. 0.03mM L-Mimosine | 1.314 | -19.49 to 22.12 | No | ns | 0.9872 |  |
|  | 0.01mM L-Mimosine vs. 0.03mM L-Mimosine | 0.7985 | -20.01 to 21.61 | No | ns | 0.9953 |  |
|  |  |  |  |  |  |  |  |
|  | Row 8 |  |  |  |  |  |  |
|  | 0mM L-Mimosine vs. 0.01mM L-Mimosine | 0.2985 | -20.51 to 21.11 | No | ns | 0.9993 |  |
|  | 0mM L-Mimosine vs. 0.03mM L-Mimosine | 1.186 | -19.62 to 21.99 | No | ns | 0.9896 |  |
|  | 0.01mM L-Mimosine vs. 0.03mM L-Mimosine | 0.8874 | -19.92 to 21.70 | No | ns | 0.9942 |  |
|  |  |  |  |  |  |  |  |
| SK-N-AS -Mimosine 48h | **Figure 6e** |  |  |  |  |  |  |
|  | **Tukey's multiple comparisons test** | Mean Diff. | 95.00% CI of diff. | Significant? | Summary | Adjusted P Value |  |
|  |  |  |  |  |  |  |  |
|  | Row 1 |  |  |  |  |  |  |
|  | 0mM L-Mimosine vs. 0.01mM L-Mimosine | -9.919 | -28.68 to 8.843 | No | ns | 0.4194 |  |
|  | 0mM L-Mimosine vs. 0.03mM L-Mimosine | -4.05 | -22.81 to 14.71 | No | ns | 0.8635 |  |
|  | 0.01mM L-Mimosine vs. 0.03mM L-Mimosine | 5.869 | -12.89 to 24.63 | No | ns | 0.7355 |  |
|  |  |  |  |  |  |  |  |
|  | Row 2 |  |  |  |  |  |  |
|  | 0mM L-Mimosine vs. 0.01mM L-Mimosine | 0.7606 | -18.00 to 19.52 | No | ns | 0.9948 |  |
|  | 0mM L-Mimosine vs. 0.03mM L-Mimosine | -3.565 | -22.33 to 15.20 | No | ns | 0.8925 |  |
|  | 0.01mM L-Mimosine vs. 0.03mM L-Mimosine | -4.325 | -23.09 to 14.44 | No | ns | 0.8459 |  |
|  |  |  |  |  |  |  |  |
|  | Row 3 |  |  |  |  |  |  |
|  | 0mM L-Mimosine vs. 0.01mM L-Mimosine | -7.162 | -25.92 to 11.60 | No | ns | 0.6336 |  |
|  | 0mM L-Mimosine vs. 0.03mM L-Mimosine | -6.507 | -25.27 to 12.25 | No | ns | 0.6857 |  |
|  | 0.01mM L-Mimosine vs. 0.03mM L-Mimosine | 0.6541 | -18.11 to 19.42 | No | ns | 0.9962 |  |
|  |  |  |  |  |  |  |  |
|  | Row 4 |  |  |  |  |  |  |
|  | 0mM L-Mimosine vs. 0.01mM L-Mimosine | 6.118 | -12.64 to 24.88 | No | ns | 0.7162 |  |
|  | 0mM L-Mimosine vs. 0.03mM L-Mimosine | 1.576 | -17.19 to 20.34 | No | ns | 0.978 |  |
|  | 0.01mM L-Mimosine vs. 0.03mM L-Mimosine | -4.542 | -23.30 to 14.22 | No | ns | 0.8315 |  |
|  |  |  |  |  |  |  |  |
|  | Row 5 |  |  |  |  |  |  |
|  | 0mM L-Mimosine vs. 0.01mM L-Mimosine | 5.483 | -13.28 to 24.24 | No | ns | 0.7647 |  |
|  | 0mM L-Mimosine vs. 0.03mM L-Mimosine | 6.363 | -12.40 to 25.13 | No | ns | 0.6971 |  |
|  | 0.01mM L-Mimosine vs. 0.03mM L-Mimosine | 0.8801 | -17.88 to 19.64 | No | ns | 0.9931 |  |
|  |  |  |  |  |  |  |  |
|  | Row 6 |  |  |  |  |  |  |
|  | 0mM L-Mimosine vs. 0.01mM L-Mimosine | -1.1 | -19.86 to 17.66 | No | ns | 0.9892 |  |
|  | 0mM L-Mimosine vs. 0.03mM L-Mimosine | 5.436 | -13.33 to 24.20 | No | ns | 0.7681 |  |
|  | 0.01mM L-Mimosine vs. 0.03mM L-Mimosine | 6.536 | -12.23 to 25.30 | No | ns | 0.6834 |  |
|  |  |  |  |  |  |  |  |
|  | Row 7 |  |  |  |  |  |  |
|  | 0mM L-Mimosine vs. 0.01mM L-Mimosine | -3.094 | -21.86 to 15.67 | No | ns | 0.9179 |  |
|  | 0mM L-Mimosine vs. 0.03mM L-Mimosine | 1.163 | -17.60 to 19.93 | No | ns | 0.9879 |  |
|  | 0.01mM L-Mimosine vs. 0.03mM L-Mimosine | 4.257 | -14.51 to 23.02 | No | ns | 0.8504 |  |
|  |  |  |  |  |  |  |  |
|  | Row 8 |  |  |  |  |  |  |
|  | 0mM L-Mimosine vs. 0.01mM L-Mimosine | 1.86 | -16.90 to 20.62 | No | ns | 0.9695 |  |
|  | 0mM L-Mimosine vs. 0.03mM L-Mimosine | -4.94 | -23.70 to 13.82 | No | ns | 0.8041 |  |
|  | 0.01mM L-Mimosine vs. 0.03mM L-Mimosine | -6.8 | -25.56 to 11.96 | No | ns | 0.6625 |  |
|  |  |  |  |  |  |  |  |
| HSC-3 L-Mimosine 48h | **Figure 6F** |  |  |  |  |  |  |
|  | **Tukey's multiple comparisons test** | Predicted (LS) mean diff. | 95.00% CI of diff. | Significant? | Summary | Adjusted P Value |  |
|  |  |  |  |  |  |  |  |
|  | Row 1 |  |  |  |  |  |  |
|  | 0mM L-Mimosine vs. 0.01mM L-Mimosine | 2.222 | -23.35 to 27.79 | No | ns | 0.9755 |  |
|  | 0mM L-Mimosine vs. 0.03mM L-Mimosine | 62.97 | 37.40 to 88.54 | Yes | **** | <0.0001 |  |
|  | 0.01mM L-Mimosine vs. 0.03mM L-Mimosine | 60.75 | 37.88 to 83.62 | Yes | **** | <0.0001 |  |
|  |  |  |  |  |  |  |  |
|  | Row 2 |  |  |  |  |  |  |
|  | 0mM L-Mimosine vs. 0.01mM L-Mimosine | 10.93 | -23.37 to 45.24 | No | ns | 0.7186 |  |
|  | 0mM L-Mimosine vs. 0.03mM L-Mimosine | 61.23 | 28.89 to 93.57 | Yes | *** | 0.0001 |  |
|  | 0.01mM L-Mimosine vs. 0.03mM L-Mimosine | 50.29 | 24.73 to 75.86 | Yes | **** | <0.0001 |  |
|  |  |  |  |  |  |  |  |
|  | Row 3 |  |  |  |  |  |  |
|  | 0mM L-Mimosine vs. 0.01mM L-Mimosine | 23.84 | -1.726 to 49.41 | No | ns | 0.0718 |  |
|  | 0mM L-Mimosine vs. 0.03mM L-Mimosine | 77.04 | 51.47 to 102.6 | Yes | **** | <0.0001 |  |
|  | 0.01mM L-Mimosine vs. 0.03mM L-Mimosine | 53.19 | 30.32 to 76.07 | Yes | **** | <0.0001 |  |
|  |  |  |  |  |  |  |  |
|  | Row 4 |  |  |  |  |  |  |
|  | 0mM L-Mimosine vs. 0.01mM L-Mimosine | -2.873 | -30.88 to 25.14 | No | ns | 0.9661 |  |
|  | 0mM L-Mimosine vs. 0.03mM L-Mimosine | 48.18 | 22.61 to 73.75 | Yes | *** | 0.0001 |  |
|  | 0.01mM L-Mimosine vs. 0.03mM L-Mimosine | 51.06 | 25.49 to 76.63 | Yes | **** | <0.0001 |  |
|  |  |  |  |  |  |  |  |
|  | Row 5 |  |  |  |  |  |  |
|  | 0mM L-Mimosine vs. 0.01mM L-Mimosine | -4.721 | -30.29 to 20.85 | No | ns | 0.8943 |  |
|  | 0mM L-Mimosine vs. 0.03mM L-Mimosine | 29.06 | 3.487 to 54.63 | Yes | * | 0.0229 |  |
|  | 0.01mM L-Mimosine vs. 0.03mM L-Mimosine | 33.78 | 10.91 to 56.65 | Yes | ** | 0.0026 |  |
|  |  |  |  |  |  |  |  |
|  | Row 6 |  |  |  |  |  |  |
|  | 0mM L-Mimosine vs. 0.01mM L-Mimosine | 4.562 | -21.01 to 30.13 | No | ns | 0.901 |  |
|  | 0mM L-Mimosine vs. 0.03mM L-Mimosine | 27.73 | 2.164 to 53.30 | Yes | * | 0.031 |  |
|  | 0.01mM L-Mimosine vs. 0.03mM L-Mimosine | 23.17 | 0.3011 to 46.04 | Yes | * | 0.0465 |  |
|  |  |  |  |  |  |  |  |
|  | Row 7 |  |  |  |  |  |  |
|  | 0mM L-Mimosine vs. 0.01mM L-Mimosine | -3.622 | -29.19 to 21.95 | No | ns | 0.9363 |  |
|  | 0mM L-Mimosine vs. 0.03mM L-Mimosine | 13.62 | -11.95 to 39.19 | No | ns | 0.4037 |  |
|  | 0.01mM L-Mimosine vs. 0.03mM L-Mimosine | 17.25 | -5.624 to 40.12 | No | ns | 0.1705 |  |
|  |  |  |  |  |  |  |  |
|  | Row 8 |  |  |  |  |  |  |
|  | 0mM L-Mimosine vs. 0.01mM L-Mimosine | 0.7529 | -24.82 to 26.32 | No | ns | 0.9972 |  |
|  | 0mM L-Mimosine vs. 0.03mM L-Mimosine | 5.007 | -20.56 to 30.58 | No | ns | 0.882 |  |
|  | 0.01mM L-Mimosine vs. 0.03mM L-Mimosine | 4.254 | -18.62 to 27.12 | No | ns | 0.8929 |  |
|  |  |  |  |  |  |  |  |
| **Flow Cytometry** | **Figure 6G** |  |  |  |  |  |  |
|  | **Column B** | **Cisplatin** |  |  |  |  |  |
|  | **vs.** | **vs.** |  |  |  |  |  |
|  | **Column A** | **Control** |  |  |  |  |  |
|  |  |  |  |  |  |  |  |
|  | Unpaired t test |  |  |  |  |  |  |
|  | P value | 0.0258 |  |  |  |  |  |
|  | P value summary | * |  |  |  |  |  |
|  | Significantly different (P < 0.05)? | Yes |  |  |  |  |  |
|  | One- or two-tailed P value? | Two-tailed |  |  |  |  |  |
|  | t, df | t=2.943, df=6 |  |  |  |  |  |
|  |  |  |  |  |  |  |  |
|  | How big is the difference? |  |  |  |  |  |  |
|  | Mean of column A | 1 |  |  |  |  |  |
|  | Mean of column B | 3.279 |  |  |  |  |  |
|  | Difference between means (B - A) ± SEM | 2.279 ± 0.7742 |  |  |  |  |  |
|  | 95% confidence interval | 0.3842 to 4.173 |  |  |  |  |  |
|  | R squared (eta squared) | 0.5908 |  |  |  |  |  |
|  |  |  |  |  |  |  |  |
|  | F test to compare variances |  |  |  |  |  |  |
|  | F, DFn, Dfd | Infinity, 3, 3 |  |  |  |  |  |
|  | P value | <0.0001 |  |  |  |  |  |
|  | P value summary | **** |  |  |  |  |  |
|  | Significantly different (P < 0.05)? | Yes |  |  |  |  |  |
|  |  |  |  |  |  |  |  |
|  | **Column C** | **Cisplatin + 0.03mM Compound X** | |  |  |  |  |
|  | **vs.** | **vs.** |  |  |  |  |  |
|  | **Column A** | **Control** |  |  |  |  |  |
|  |  |  |  |  |  |  |  |
|  | Unpaired t test |  |  |  |  |  |  |
|  | P value | 0.0133 |  |  |  |  |  |
|  | P value summary | * |  |  |  |  |  |
|  | Significantly different (P < 0.05)? | Yes |  |  |  |  |  |
|  | One- or two-tailed P value? | Two-tailed |  |  |  |  |  |
|  | t, df | t=3.470, df=6 |  |  |  |  |  |
|  |  |  |  |  |  |  |  |
|  | How big is the difference? |  |  |  |  |  |  |
|  | Mean of column A | 1 |  |  |  |  |  |
|  | Mean of column C | 5.175 |  |  |  |  |  |
|  | Difference between means (C - A) ± SEM | 4.175 ± 1.203 |  |  |  |  |  |
|  | 95% confidence interval | 1.231 to 7.118 |  |  |  |  |  |
|  | R squared (eta squared) | 0.6674 |  |  |  |  |  |
|  |  |  |  |  |  |  |  |
|  | F test to compare variances |  |  |  |  |  |  |
|  | F, DFn, Dfd | Infinity, 3, 3 |  |  |  |  |  |
|  | P value | <0.0001 |  |  |  |  |  |
|  | P value summary | **** |  |  |  |  |  |
|  | Significantly different (P < 0.05)? | Yes |  |  |  |  |  |
|  |  |  |  |  |  |  |  |
|  | **Column D** | **Cisplatin + 0.03mM Compound Y** | |  |  |  |  |
|  | **vs.** | **vs.** |  |  |  |  |  |
|  | **Column A** | **Control** |  |  |  |  |  |
|  |  |  |  |  |  |  |  |
|  | Unpaired t test |  |  |  |  |  |  |
|  | P value | <0.0001 |  |  |  |  |  |
|  | P value summary | **** |  |  |  |  |  |
|  | Significantly different (P < 0.05)? | Yes |  |  |  |  |  |
|  | One- or two-tailed P value? | Two-tailed |  |  |  |  |  |
|  | t, df | t=15.78, df=6 |  |  |  |  |  |
|  |  |  |  |  |  |  |  |
|  | How big is the difference? |  |  |  |  |  |  |
|  | Mean of column A | 1 |  |  |  |  |  |
|  | Mean of column D | 3.855 |  |  |  |  |  |
|  | Difference between means (D - A) ± SEM | 2.855 ± 0.1810 |  |  |  |  |  |
|  | 95% confidence interval | 2.413 to 3.298 |  |  |  |  |  |
|  | R squared (eta squared) | 0.9765 |  |  |  |  |  |
|  |  |  |  |  |  |  |  |
|  | F test to compare variances |  |  |  |  |  |  |
|  | F, DFn, Dfd | Infinity, 3, 3 |  |  |  |  |  |
|  | P value | <0.0001 |  |  |  |  |  |
|  | P value summary | **** |  |  |  |  |  |
|  | Significantly different (P < 0.05)? | Yes |  |  |  |  |  |
|  |  |  |  |  |  |  |  |
| SK-N-AS H2AX | **Figure 6H** |  |  |  |  |  |  |
|  | **Dunn's multiple comparisons test** | Mean rank diff. | Significant? | Summary | Adjusted P Value |  |  |
|  | Control vs. Cisplatin (0.01mM) | -367.5 | Yes | **** | <0.0001 |  |  |
|  | Control vs. Cisplatin (0.01mM) + Dopamine (0.03mM) | -330 | Yes | **** | <0.0001 |  |  |
|  | Control vs. Cisplatin (0.01mM) + L-Mimosine (0.03mM) | -446.5 | Yes | **** | <0.0001 |  |  |
|  | Cisplatin (0.01mM) vs. Cisplatin (0.01mM) + Dopamine (0.03mM) | 37.54 | No | ns | 0.9256 |  |  |
|  | Cisplatin (0.01mM) vs. Cisplatin (0.01mM) + L-Mimosine (0.03mM) | -78.98 | Yes | * | 0.0254 |  |  |
|  | Cisplatin (0.01mM) + Dopamine (0.03mM) vs. Cisplatin (0.01mM) + L-Mimosine (0.03mM) | -116.5 | Yes | **** | <0.0001 |  |  |
|  |  |  |  |  |  |  |  |
|  |  |  |  |  |  |  |  |
| HSC3 H2AX | **Figure 6I** |  |  |  |  |  |  |
|  | **Dunn's multiple comparisons test** | Mean rank diff. | Significant? | Summary | Adjusted P Value |  |  |
|  | Control vs. Cisplatin (0.01mM) | -727.1 | Yes | **** | <0.0001 |  |  |
|  | Control vs. Cisplatin (0.01mM) + Dopamine (0.03mM) | -594.9 | Yes | **** | <0.0001 |  |  |
|  | Control vs. Cisplatin (0.01mM) + L-Mimosine (0.03mM) | -431.3 | Yes | **** | <0.0001 |  |  |
|  | Cisplatin (0.01mM) vs. Cisplatin (0.01mM) + Dopamine (0.03mM) | 132.2 | Yes | ** | 0.001 |  |  |
|  | Cisplatin (0.01mM) vs. Cisplatin (0.01mM) + L-Mimosine (0.03mM) | 295.8 | Yes | **** | <0.0001 |  |  |
|  | Cisplatin (0.01mM) + Dopamine (0.03mM) vs. Cisplatin (0.01mM) + L-Mimosine (0.03mM) | 163.7 | Yes | **** | <0.0001 |  |  |
|  |  |  |  |  |  |  |  |
|  |  |  |  |  |  |  |  |
| ImageJ Inulin Analysis | **Figure 5 - Supplement 1a** |  |  |  |  |  |  |
|  | Unpaired t test |  |  |  |  |  |  |
|  | P value | 0.0311 |  |  |  |  |  |
|  | P value summary | * |  |  |  |  |  |
|  | Significantly different (P < 0.05)? | Yes |  |  |  |  |  |
|  | One- or two-tailed P value? | Two-tailed |  |  |  |  |  |
|  | t, df | t=2.396, df=14 |  |  |  |  |  |
|  |  |  |  |  |  |  |  |
| Biosorter Analysis Optimization | **Figure 5 - Figure Supplement 1b** |  |  |  |  |  |  |
|  | Tukey's multiple comparisons test | Predicted (LS) mean diff. | 95.00% CI of diff. | Significant? | Summary | Adjusted P Value |  |
|  |  |  |  |  |  |  |  |
|  | 0:Control vs. 0:Cisplatin | -127.8 | -1266 to 1010 | No | ns | 0.9914 |  |
|  | 0:Control vs. 2:Control | 3176 | 2121 to 4231 | Yes | **** | <0.0001 |  |
|  | 0:Control vs. 2:Cisplatin | 464.2 | -748.5 to 1677 | No | ns | 0.7549 |  |
|  | 0:Cisplatin vs. 2:Control | 3304 | 2131 to 4477 | Yes | **** | <0.0001 |  |
|  | 0:Cisplatin vs. 2:Cisplatin | 591.9 | -724.8 to 1909 | No | ns | 0.6504 |  |
|  | 2:Control vs. 2:Cisplatin | -2712 | -3957 to -1466 | Yes | **** | <0.0001 |  |
|  |  |  |  |  |  |  |  |
| Heart Rate Analysis | **Figure 5 - Figure Supplement 1c** |  |  |  |  |  |  |
|  | Column B | Cisplatin (0.125mM) | |  |  |  |  |
|  | vs. | vs. |  |  |  |  |  |
|  | Column A | Control |  |  |  |  |  |
|  |  |  |  |  |  |  |  |
|  | Unpaired t test |  |  |  |  |  |  |
|  | P value | 0.6363 |  |  |  |  |  |
|  | P value summary | ns |  |  |  |  |  |
|  | Significantly different (P < 0.05)? | No |  |  |  |  |  |
|  | One- or two-tailed P value? | Two-tailed |  |  |  |  |  |
|  | t, df | t=0.4790, df=24 |  |  |  |  |  |
|  |  |  |  |  |  |  |  |
|  | How big is the difference? |  |  |  |  |  |  |
|  | Mean of column A | 148 |  |  |  |  |  |
|  | Mean of column B | 144 |  |  |  |  |  |
|  | Difference between means (B - A) ± SEM | -4.000 ± 8.351 |  |  |  |  |  |
|  | 95% confidence interval | -21.24 to 13.24 |  |  |  |  |  |
|  | R squared (eta squared) | 0.009468 |  |  |  |  |  |
|  |  |  |  |  |  |  |  |
|  | F test to compare variances |  |  |  |  |  |  |
|  | F, DFn, Dfd | 1.982, 12, 12 |  |  |  |  |  |
|  | P value | 0.2501 |  |  |  |  |  |
|  | P value summary | ns |  |  |  |  |  |
|  | Significantly different (P < 0.05)? | No |  |  |  |  |  |
|  |  |  |  |  |  |  |  |
|  |  |  |  |  |  |  |  |
| LAN5 Dopamine 24h | **Figure 6 - Figure Supplement 1a** |  |  |  |  |  |  |
|  | **Tukey's multiple comparisons test** | Predicted (LS) mean diff. | 95.00% CI of diff. | Significant? | Summary | Adjusted P Value |  |
|  |  |  |  |  |  |  |  |
|  | Row 1 |  |  |  |  |  |  |
|  | 0mM Dopamine vs. 0.01mM Dopamine | 22.58 | 5.445 to 39.71 | Yes | ** | 0.0073 |  |
|  | 0mM Dopamine vs. 0.03mM Dopamine | 87.81 | 68.66 to 107.0 | Yes | **** | <0.0001 |  |
|  | 0.01mM Dopamine vs. 0.03mM Dopamine | 65.23 | 46.08 to 84.38 | Yes | **** | <0.0001 |  |
|  |  |  |  |  |  |  |  |
|  | Row 2 |  |  |  |  |  |  |
|  | 0mM Dopamine vs. 0.01mM Dopamine | 16.66 | -0.4667 to 33.79 | No | ns | 0.058 |  |
|  | 0mM Dopamine vs. 0.03mM Dopamine | 82.64 | 63.48 to 101.8 | Yes | **** | <0.0001 |  |
|  | 0.01mM Dopamine vs. 0.03mM Dopamine | 65.97 | 46.82 to 85.13 | Yes | **** | <0.0001 |  |
|  |  |  |  |  |  |  |  |
|  | Row 3 |  |  |  |  |  |  |
|  | 0mM Dopamine vs. 0.01mM Dopamine | 31.63 | 14.50 to 48.77 | Yes | *** | 0.0002 |  |
|  | 0mM Dopamine vs. 0.03mM Dopamine | 93.92 | 74.77 to 113.1 | Yes | **** | <0.0001 |  |
|  | 0.01mM Dopamine vs. 0.03mM Dopamine | 62.29 | 43.14 to 81.44 | Yes | **** | <0.0001 |  |
|  |  |  |  |  |  |  |  |
|  | Row 4 |  |  |  |  |  |  |
|  | 0mM Dopamine vs. 0.01mM Dopamine | 18.23 | 1.100 to 35.36 | Yes | * | 0.0348 |  |
|  | 0mM Dopamine vs. 0.03mM Dopamine | 78.95 | 59.80 to 98.10 | Yes | **** | <0.0001 |  |
|  | 0.01mM Dopamine vs. 0.03mM Dopamine | 60.72 | 41.56 to 79.87 | Yes | **** | <0.0001 |  |
|  |  |  |  |  |  |  |  |
|  | Row 5 |  |  |  |  |  |  |
|  | 0mM Dopamine vs. 0.01mM Dopamine | 20.01 | 2.880 to 37.14 | Yes | * | 0.0188 |  |
|  | 0mM Dopamine vs. 0.03mM Dopamine | 74.9 | 55.75 to 94.05 | Yes | **** | <0.0001 |  |
|  | 0.01mM Dopamine vs. 0.03mM Dopamine | 54.89 | 35.74 to 74.04 | Yes | **** | <0.0001 |  |
|  |  |  |  |  |  |  |  |
|  | Row 6 |  |  |  |  |  |  |
|  | 0mM Dopamine vs. 0.01mM Dopamine | 5.166 | -11.96 to 22.30 | No | ns | 0.7449 |  |
|  | 0mM Dopamine vs. 0.03mM Dopamine | 49.81 | 30.66 to 68.97 | Yes | **** | <0.0001 |  |
|  | 0.01mM Dopamine vs. 0.03mM Dopamine | 44.65 | 25.50 to 63.80 | Yes | **** | <0.0001 |  |
|  |  |  |  |  |  |  |  |
|  | Row 7 |  |  |  |  |  |  |
|  | 0mM Dopamine vs. 0.01mM Dopamine | 3.143 | -13.99 to 20.27 | No | ns | 0.8962 |  |
|  | 0mM Dopamine vs. 0.03mM Dopamine | 28.35 | 9.200 to 47.51 | Yes | ** | 0.0024 |  |
|  | 0.01mM Dopamine vs. 0.03mM Dopamine | 25.21 | 6.057 to 44.36 | Yes | ** | 0.0073 |  |
|  |  |  |  |  |  |  |  |
|  | Row 8 |  |  |  |  |  |  |
|  | 0mM Dopamine vs. 0.01mM Dopamine | -4.073 | -21.20 to 13.06 | No | ns | 0.8323 |  |
|  | 0mM Dopamine vs. 0.03mM Dopamine | 16.25 | -2.898 to 35.41 | No | ns | 0.11 |  |
|  | 0.01mM Dopamine vs. 0.03mM Dopamine | 20.33 | 1.175 to 39.48 | Yes | * | 0.0354 |  |
|  |  |  |  |  |  |  |  |
| LAN5 L-Mimosine 24h | **Figure 6 - Figure Supplement 1b** |  |  |  |  |  |  |
|  | **Tukey's multiple comparisons test** | Mean Diff. | 95.00% CI of diff. | Significant? | Summary | Adjusted P Value |  |
|  |  |  |  |  |  |  |  |
|  | Row 1 |  |  |  |  |  |  |
|  | 0mM L-Mimosine vs. 0.01mM L-Mimosine | -3.127 | -34.41 to 28.16 | No | ns | 0.9683 |  |
|  | 0mM L-Mimosine vs. 0.03mM L-Mimosine | -0.7394 | -32.03 to 30.55 | No | ns | 0.9982 |  |
|  | 0.01mM L-Mimosine vs. 0.03mM L-Mimosine | 2.387 | -28.90 to 33.67 | No | ns | 0.9814 |  |
|  |  |  |  |  |  |  |  |
|  | Row 2 |  |  |  |  |  |  |
|  | 0mM L-Mimosine vs. 0.01mM L-Mimosine | 18.04 | -13.24 to 49.33 | No | ns | 0.3517 |  |
|  | 0mM L-Mimosine vs. 0.03mM L-Mimosine | 16.26 | -15.02 to 47.55 | No | ns | 0.4261 |  |
|  | 0.01mM L-Mimosine vs. 0.03mM L-Mimosine | -1.78 | -33.07 to 29.51 | No | ns | 0.9896 |  |
|  |  |  |  |  |  |  |  |
|  | Row 3 |  |  |  |  |  |  |
|  | 0mM L-Mimosine vs. 0.01mM L-Mimosine | 11.14 | -20.15 to 42.42 | No | ns | 0.6673 |  |
|  | 0mM L-Mimosine vs. 0.03mM L-Mimosine | 13.31 | -17.98 to 44.60 | No | ns | 0.5625 |  |
|  | 0.01mM L-Mimosine vs. 0.03mM L-Mimosine | 2.174 | -29.11 to 33.46 | No | ns | 0.9846 |  |
|  |  |  |  |  |  |  |  |
|  | Row 4 |  |  |  |  |  |  |
|  | 0mM L-Mimosine vs. 0.01mM L-Mimosine | 11.45 | -19.83 to 42.74 | No | ns | 0.6521 |  |
|  | 0mM L-Mimosine vs. 0.03mM L-Mimosine | 6.998 | -24.29 to 38.28 | No | ns | 0.8516 |  |
|  | 0.01mM L-Mimosine vs. 0.03mM L-Mimosine | -4.455 | -35.74 to 26.83 | No | ns | 0.9368 |  |
|  |  |  |  |  |  |  |  |
|  | Row 5 |  |  |  |  |  |  |
|  | 0mM L-Mimosine vs. 0.01mM L-Mimosine | 3.589 | -27.70 to 34.88 | No | ns | 0.9585 |  |
|  | 0mM L-Mimosine vs. 0.03mM L-Mimosine | 1.279 | -30.01 to 32.57 | No | ns | 0.9946 |  |
|  | 0.01mM L-Mimosine vs. 0.03mM L-Mimosine | -2.311 | -33.60 to 28.98 | No | ns | 0.9826 |  |
|  |  |  |  |  |  |  |  |
|  | Row 6 |  |  |  |  |  |  |
|  | 0mM L-Mimosine vs. 0.01mM L-Mimosine | 4.879 | -26.41 to 36.17 | No | ns | 0.9247 |  |
|  | 0mM L-Mimosine vs. 0.03mM L-Mimosine | 8.725 | -22.56 to 40.01 | No | ns | 0.7794 |  |
|  | 0.01mM L-Mimosine vs. 0.03mM L-Mimosine | 3.845 | -27.44 to 35.13 | No | ns | 0.9525 |  |
|  |  |  |  |  |  |  |  |
|  | Row 7 |  |  |  |  |  |  |
|  | 0mM L-Mimosine vs. 0.01mM L-Mimosine | 1.147 | -30.14 to 32.43 | No | ns | 0.9957 |  |
|  | 0mM L-Mimosine vs. 0.03mM L-Mimosine | -0.07038 | -31.36 to 31.22 | No | ns | >0.9999 |  |
|  | 0.01mM L-Mimosine vs. 0.03mM L-Mimosine | -1.218 | -32.50 to 30.07 | No | ns | 0.9951 |  |
|  |  |  |  |  |  |  |  |
|  | Row 8 |  |  |  |  |  |  |
|  | 0mM L-Mimosine vs. 0.01mM L-Mimosine | -0.426 | -31.71 to 30.86 | No | ns | 0.9994 |  |
|  | 0mM L-Mimosine vs. 0.03mM L-Mimosine | 0.906 | -30.38 to 32.19 | No | ns | 0.9973 |  |
|  | 0.01mM L-Mimosine vs. 0.03mM L-Mimosine | 1.332 | -29.95 to 32.62 | No | ns | 0.9942 |  |
|  |  |  |  |  |  |  |  |
| SK-N-AS Dopamine 24hr | **Figure 6 - Figure Supplement 1c** |  |  |  |  |  |  |
|  | **Tukey's multiple comparisons test** | Mean Diff. | 95.00% CI of diff. | Significant? | Summary | Adjusted P Value |  |
|  |  |  |  |  |  |  |  |
|  | Row 1 |  |  |  |  |  |  |
|  | 0mM Dopamine vs. 0.01mM Dopamine | 1.061 | -42.44 to 44.56 | No | ns | 0.9981 |  |
|  | 0mM Dopamine vs. 0.03mM Dopamine | 56.06 | 12.56 to 99.57 | Yes | ** | 0.0085 |  |
|  | 0.01mM Dopamine vs. 0.03mM Dopamine | 55 | 11.50 to 98.50 | Yes | ** | 0.01 |  |
|  |  |  |  |  |  |  |  |
|  | Row 2 |  |  |  |  |  |  |
|  | 0mM Dopamine vs. 0.01mM Dopamine | 2.266 | -41.24 to 45.77 | No | ns | 0.9913 |  |
|  | 0mM Dopamine vs. 0.03mM Dopamine | 55.92 | 12.42 to 99.42 | Yes | ** | 0.0087 |  |
|  | 0.01mM Dopamine vs. 0.03mM Dopamine | 53.65 | 10.15 to 97.15 | Yes | * | 0.0122 |  |
|  |  |  |  |  |  |  |  |
|  | Row 3 |  |  |  |  |  |  |
|  | 0mM Dopamine vs. 0.01mM Dopamine | 3.447 | -40.05 to 46.95 | No | ns | 0.98 |  |
|  | 0mM Dopamine vs. 0.03mM Dopamine | 51.16 | 7.662 to 94.67 | Yes | * | 0.0176 |  |
|  | 0.01mM Dopamine vs. 0.03mM Dopamine | 47.72 | 4.215 to 91.22 | Yes | * | 0.0286 |  |
|  |  |  |  |  |  |  |  |
|  | Row 4 |  |  |  |  |  |  |
|  | 0mM Dopamine vs. 0.01mM Dopamine | 7.899 | -35.60 to 51.40 | No | ns | 0.8994 |  |
|  | 0mM Dopamine vs. 0.03mM Dopamine | 65.38 | 21.88 to 108.9 | Yes | ** | 0.0019 |  |
|  | 0.01mM Dopamine vs. 0.03mM Dopamine | 57.48 | 13.98 to 101.0 | Yes | ** | 0.0068 |  |
|  |  |  |  |  |  |  |  |
|  | Row 5 |  |  |  |  |  |  |
|  | 0mM Dopamine vs. 0.01mM Dopamine | 11.03 | -32.48 to 54.53 | No | ns | 0.8138 |  |
|  | 0mM Dopamine vs. 0.03mM Dopamine | 56.84 | 13.34 to 100.3 | Yes | ** | 0.0076 |  |
|  | 0.01mM Dopamine vs. 0.03mM Dopamine | 45.81 | 2.312 to 89.32 | Yes | * | 0.0369 |  |
|  |  |  |  |  |  |  |  |
|  | Row 6 |  |  |  |  |  |  |
|  | 0mM Dopamine vs. 0.01mM Dopamine | 3.721 | -39.78 to 47.22 | No | ns | 0.9767 |  |
|  | 0mM Dopamine vs. 0.03mM Dopamine | 58.01 | 14.50 to 101.5 | Yes | ** | 0.0063 |  |
|  | 0.01mM Dopamine vs. 0.03mM Dopamine | 54.28 | 10.78 to 97.79 | Yes | * | 0.0111 |  |
|  |  |  |  |  |  |  |  |
|  | Row 7 |  |  |  |  |  |  |
|  | 0mM Dopamine vs. 0.01mM Dopamine | 10.69 | -32.82 to 54.19 | No | ns | 0.824 |  |
|  | 0mM Dopamine vs. 0.03mM Dopamine | 60.77 | 17.27 to 104.3 | Yes | ** | 0.0041 |  |
|  | 0.01mM Dopamine vs. 0.03mM Dopamine | 50.09 | 6.587 to 93.59 | Yes | * | 0.0205 |  |
|  |  |  |  |  |  |  |  |
|  | Row 8 |  |  |  |  |  |  |
|  | 0mM Dopamine vs. 0.01mM Dopamine | 10.74 | -32.76 to 54.24 | No | ns | 0.8223 |  |
|  | 0mM Dopamine vs. 0.03mM Dopamine | 65.18 | 21.68 to 108.7 | Yes | ** | 0.002 |  |
|  | 0.01mM Dopamine vs. 0.03mM Dopamine | 54.44 | 10.94 to 97.94 | Yes | * | 0.0109 |  |
|  |  |  |  |  |  |  |  |
| SK-N-AS L-Mimosine 24h | **Figure 6 - Figure Supplement 1d** |  |  |  |  |  |  |
|  | **Tukey's multiple comparisons test** | Mean Diff. | 95.00% CI of diff. | Significant? | Summary | Adjusted P Value |  |
|  |  |  |  |  |  |  |  |
|  | Row 1 |  |  |  |  |  |  |
|  | 0mM L-Mimosine vs. 0.01mM L-Mimosine | 23.64 | -16.45 to 63.74 | No | ns | 0.3215 |  |
|  | 0mM L-Mimosine vs. 0.03mM L-Mimosine | 5.747 | -34.35 to 45.84 | No | ns | 0.932 |  |
|  | 0.01mM L-Mimosine vs. 0.03mM L-Mimosine | -17.9 | -57.99 to 22.20 | No | ns | 0.5146 |  |
|  |  |  |  |  |  |  |  |
|  | Row 2 |  |  |  |  |  |  |
|  | 0mM L-Mimosine vs. 0.01mM L-Mimosine | 10.34 | -29.75 to 50.44 | No | ns | 0.7974 |  |
|  | 0mM L-Mimosine vs. 0.03mM L-Mimosine | 15.37 | -24.72 to 55.47 | No | ns | 0.6102 |  |
|  | 0.01mM L-Mimosine vs. 0.03mM L-Mimosine | 5.03 | -35.06 to 45.12 | No | ns | 0.9475 |  |
|  |  |  |  |  |  |  |  |
|  | Row 3 |  |  |  |  |  |  |
|  | 0mM L-Mimosine vs. 0.01mM L-Mimosine | 29.34 | -10.76 to 69.43 | No | ns | 0.1823 |  |
|  | 0mM L-Mimosine vs. 0.03mM L-Mimosine | 37.62 | -2.473 to 77.72 | No | ns | 0.0688 |  |
|  | 0.01mM L-Mimosine vs. 0.03mM L-Mimosine | 8.286 | -31.81 to 48.38 | No | ns | 0.8643 |  |
|  |  |  |  |  |  |  |  |
|  | Row 4 |  |  |  |  |  |  |
|  | 0mM L-Mimosine vs. 0.01mM L-Mimosine | 9.808 | -30.29 to 49.90 | No | ns | 0.8156 |  |
|  | 0mM L-Mimosine vs. 0.03mM L-Mimosine | 14.84 | -25.25 to 54.94 | No | ns | 0.6304 |  |
|  | 0.01mM L-Mimosine vs. 0.03mM L-Mimosine | 5.036 | -35.06 to 45.13 | No | ns | 0.9473 |  |
|  |  |  |  |  |  |  |  |
|  | Row 5 |  |  |  |  |  |  |
|  | 0mM L-Mimosine vs. 0.01mM L-Mimosine | -4.007 | -44.10 to 36.09 | No | ns | 0.9663 |  |
|  | 0mM L-Mimosine vs. 0.03mM L-Mimosine | -0.4222 | -40.52 to 39.67 | No | ns | 0.9996 |  |
|  | 0.01mM L-Mimosine vs. 0.03mM L-Mimosine | 3.584 | -36.51 to 43.68 | No | ns | 0.9729 |  |
|  |  |  |  |  |  |  |  |
|  | Row 6 |  |  |  |  |  |  |
|  | 0mM L-Mimosine vs. 0.01mM L-Mimosine | 0.8444 | -39.25 to 40.94 | No | ns | 0.9985 |  |
|  | 0mM L-Mimosine vs. 0.03mM L-Mimosine | 2.847 | -37.25 to 42.94 | No | ns | 0.9828 |  |
|  | 0.01mM L-Mimosine vs. 0.03mM L-Mimosine | 2.003 | -38.09 to 42.10 | No | ns | 0.9915 |  |
|  |  |  |  |  |  |  |  |
|  | Row 7 |  |  |  |  |  |  |
|  | 0mM L-Mimosine vs. 0.01mM L-Mimosine | 9.196 | -30.90 to 49.29 | No | ns | 0.8358 |  |
|  | 0mM L-Mimosine vs. 0.03mM L-Mimosine | -2.288 | -42.38 to 37.81 | No | ns | 0.9889 |  |
|  | 0.01mM L-Mimosine vs. 0.03mM L-Mimosine | -11.48 | -51.58 to 28.61 | No | ns | 0.7569 |  |
|  |  |  |  |  |  |  |  |
|  | Row 8 |  |  |  |  |  |  |
|  | 0mM L-Mimosine vs. 0.01mM L-Mimosine | 17.06 | -23.04 to 57.15 | No | ns | 0.5459 |  |
|  | 0mM L-Mimosine vs. 0.03mM L-Mimosine | 7.539 | -32.56 to 47.63 | No | ns | 0.8861 |  |
|  | 0.01mM L-Mimosine vs. 0.03mM L-Mimosine | -9.518 | -49.61 to 30.58 | No | ns | 0.8253 |  |
|  |  |  |  |  |  |  |  |
| HSC-3 Dopamine 24hr | **Figure 6 - Figure Supplement 1e** |  |  |  |  |  |  |
|  | **Tukey's multiple comparisons test** | Predicted (LS) mean diff. | 95.00% CI of diff. | Significant? | Summary | Adjusted P Value |  |
|  |  |  |  |  |  |  |  |
|  | Row 1 |  |  |  |  |  |  |
|  | mM Cisplatin vs. 0.01mM Dopamine | 8.76 | -22.86 to 40.38 | No | ns | 0.78 |  |
|  | mM Cisplatin vs. 0.03mM Dopamine | 8.602 | -23.02 to 40.22 | No | ns | 0.7869 |  |
|  | 0.01mM Dopamine vs. 0.03mM Dopamine | -0.1578 | -31.78 to 31.46 | No | ns | >0.9999 |  |
|  |  |  |  |  |  |  |  |
|  | Row 2 |  |  |  |  |  |  |
|  | mM Cisplatin vs. 0.01mM Dopamine | 20.11 | -11.51 to 51.73 | No | ns | 0.2804 |  |
|  | mM Cisplatin vs. 0.03mM Dopamine | 0.451 | -34.90 to 35.81 | No | ns | 0.9995 |  |
|  | 0.01mM Dopamine vs. 0.03mM Dopamine | -19.66 | -55.01 to 15.70 | No | ns | 0.3752 |  |
|  |  |  |  |  |  |  |  |
|  | Row 3 |  |  |  |  |  |  |
|  | mM Cisplatin vs. 0.01mM Dopamine | 16.17 | -15.45 to 47.79 | No | ns | 0.4348 |  |
|  | mM Cisplatin vs. 0.03mM Dopamine | 8.732 | -22.89 to 40.35 | No | ns | 0.7813 |  |
|  | 0.01mM Dopamine vs. 0.03mM Dopamine | -7.44 | -39.06 to 24.18 | No | ns | 0.8356 |  |
|  |  |  |  |  |  |  |  |
|  | Row 4 |  |  |  |  |  |  |
|  | mM Cisplatin vs. 0.01mM Dopamine | -11.03 | -49.76 to 27.69 | No | ns | 0.769 |  |
|  | mM Cisplatin vs. 0.03mM Dopamine | -13.81 | -52.54 to 24.92 | No | ns | 0.6638 |  |
|  | 0.01mM Dopamine vs. 0.03mM Dopamine | -2.775 | -41.50 to 35.95 | No | ns | 0.9834 |  |
|  |  |  |  |  |  |  |  |
|  | Row 5 |  |  |  |  |  |  |
|  | mM Cisplatin vs. 0.01mM Dopamine | -5.186 | -43.92 to 33.54 | No | ns | 0.9433 |  |
|  | mM Cisplatin vs. 0.03mM Dopamine | 4.138 | -34.59 to 42.87 | No | ns | 0.9635 |  |
|  | 0.01mM Dopamine vs. 0.03mM Dopamine | 9.325 | -29.40 to 48.05 | No | ns | 0.8286 |  |
|  |  |  |  |  |  |  |  |
|  | Row 6 |  |  |  |  |  |  |
|  | mM Cisplatin vs. 0.01mM Dopamine | 0.1562 | -31.47 to 31.78 | No | ns | >0.9999 |  |
|  | mM Cisplatin vs. 0.03mM Dopamine | -0.4123 | -32.03 to 31.21 | No | ns | 0.9994 |  |
|  | 0.01mM Dopamine vs. 0.03mM Dopamine | -0.5685 | -32.19 to 31.05 | No | ns | 0.9989 |  |
|  |  |  |  |  |  |  |  |
|  | Row 7 |  |  |  |  |  |  |
|  | mM Cisplatin vs. 0.01mM Dopamine | 9.697 | -21.92 to 41.32 | No | ns | 0.7379 |  |
|  | mM Cisplatin vs. 0.03mM Dopamine | 6.839 | -24.78 to 38.46 | No | ns | 0.8591 |  |
|  | 0.01mM Dopamine vs. 0.03mM Dopamine | -2.858 | -34.48 to 28.76 | No | ns | 0.9737 |  |
|  |  |  |  |  |  |  |  |
|  | Row 8 |  |  |  |  |  |  |
|  | mM Cisplatin vs. 0.01mM Dopamine | 3.672 | -27.95 to 35.29 | No | ns | 0.9571 |  |
|  | mM Cisplatin vs. 0.03mM Dopamine | 10.86 | -20.76 to 42.48 | No | ns | 0.6836 |  |
|  | 0.01mM Dopamine vs. 0.03mM Dopamine | 7.189 | -24.43 to 38.81 | No | ns | 0.8456 |  |
|  |  |  |  |  |  |  |  |
| HSC-3 L-Mimosine 24h | **Figure 6 - Figure Supplement 1f** |  |  |  |  |  |  |
|  | **Tukey's multiple comparisons test** | Mean Diff. | 95.00% CI of diff. | Significant? | Summary | Adjusted P Value |  |
|  |  |  |  |  |  |  |  |
|  | Row 1 |  |  |  |  |  |  |
|  | 0mM L-Mimosine vs. 0.01mM L-Mimosine | 14.14 | -27.52 to 55.81 | No | ns | 0.692 |  |
|  | 0mM L-Mimosine vs. 0.03mM L-Mimosine | 52.71 | 11.04 to 94.38 | Yes | ** | 0.01 |  |
|  | 0.01mM L-Mimosine vs. 0.03mM L-Mimosine | 38.57 | -3.099 to 80.24 | No | ns | 0.0749 |  |
|  |  |  |  |  |  |  |  |
|  | Row 2 |  |  |  |  |  |  |
|  | 0mM L-Mimosine vs. 0.01mM L-Mimosine | 14.37 | -27.30 to 56.04 | No | ns | 0.6838 |  |
|  | 0mM L-Mimosine vs. 0.03mM L-Mimosine | 45.22 | 3.553 to 86.89 | Yes | * | 0.0306 |  |
|  | 0.01mM L-Mimosine vs. 0.03mM L-Mimosine | 30.85 | -10.82 to 72.52 | No | ns | 0.1836 |  |
|  |  |  |  |  |  |  |  |
|  | Row 3 |  |  |  |  |  |  |
|  | 0mM L-Mimosine vs. 0.01mM L-Mimosine | 24.94 | -16.73 to 66.61 | No | ns | 0.3251 |  |
|  | 0mM L-Mimosine vs. 0.03mM L-Mimosine | 51.22 | 9.549 to 92.88 | Yes | * | 0.0126 |  |
|  | 0.01mM L-Mimosine vs. 0.03mM L-Mimosine | 26.28 | -15.39 to 67.94 | No | ns | 0.2884 |  |
|  |  |  |  |  |  |  |  |
|  | Row 4 |  |  |  |  |  |  |
|  | 0mM L-Mimosine vs. 0.01mM L-Mimosine | 17.86 | -23.81 to 59.53 | No | ns | 0.5577 |  |
|  | 0mM L-Mimosine vs. 0.03mM L-Mimosine | 52.47 | 10.80 to 94.14 | Yes | * | 0.0103 |  |
|  | 0.01mM L-Mimosine vs. 0.03mM L-Mimosine | 34.61 | -7.058 to 76.28 | No | ns | 0.121 |  |
|  |  |  |  |  |  |  |  |
|  | Row 5 |  |  |  |  |  |  |
|  | 0mM L-Mimosine vs. 0.01mM L-Mimosine | 12.07 | -29.60 to 53.74 | No | ns | 0.7643 |  |
|  | 0mM L-Mimosine vs. 0.03mM L-Mimosine | 47.16 | 5.492 to 88.83 | Yes | * | 0.0231 |  |
|  | 0.01mM L-Mimosine vs. 0.03mM L-Mimosine | 35.09 | -6.578 to 76.76 | No | ns | 0.1144 |  |
|  |  |  |  |  |  |  |  |
|  | Row 6 |  |  |  |  |  |  |
|  | 0mM L-Mimosine vs. 0.01mM L-Mimosine | 14.44 | -27.23 to 56.11 | No | ns | 0.6815 |  |
|  | 0mM L-Mimosine vs. 0.03mM L-Mimosine | 47.97 | 6.302 to 89.64 | Yes | * | 0.0205 |  |
|  | 0.01mM L-Mimosine vs. 0.03mM L-Mimosine | 33.53 | -8.136 to 75.20 | No | ns | 0.1369 |  |
|  |  |  |  |  |  |  |  |
|  | Row 7 |  |  |  |  |  |  |
|  | 0mM L-Mimosine vs. 0.01mM L-Mimosine | 5.813 | -35.85 to 47.48 | No | ns | 0.9393 |  |
|  | 0mM L-Mimosine vs. 0.03mM L-Mimosine | 38.55 | -3.113 to 80.22 | No | ns | 0.075 |  |
|  | 0.01mM L-Mimosine vs. 0.03mM L-Mimosine | 32.74 | -8.926 to 74.41 | No | ns | 0.1496 |  |
|  |  |  |  |  |  |  |  |
|  | Row 8 |  |  |  |  |  |  |
|  | 0mM L-Mimosine vs. 0.01mM L-Mimosine | 4.765 | -36.90 to 46.43 | No | ns | 0.9587 |  |
|  | 0mM L-Mimosine vs. 0.03mM L-Mimosine | 32.19 | -9.482 to 73.85 | No | ns | 0.159 |  |
|  | 0.01mM L-Mimosine vs. 0.03mM L-Mimosine | 27.42 | -14.25 to 69.09 | No | ns | 0.2591 |  |
|  |  |  |  |  |  |  |  |
|  |  |  |  |  |  |  |  |
