# Supplementary material for: The identification of dual protective agents against cisplatin-induced oto- and nephrotoxicity using the zebrafish model
Source: eLife. 2020 Jul 28;9:e56235. doi: 10.7554/eLife.56235 (PMC7470826; doi:10.7554/eLife.56235)
Supplement: Transparent reporting form [file elife-56235-transrepform.docx]

***eLife’s* transparent reporting form**

We encourage authors to provide detailed information *within their submission* to facilitate the interpretation and replication of experiments. Authors can upload supporting documentation to indicate the use of appropriate reporting guidelines for health-related research (see [EQUATOR Network](http://www.equator-network.org/%20)), life science research (see the [BioSharing Information Resource](https://biosharing.org/)), or the [ARRIVE guidelines](http://www.plosbiology.org/article/info:doi/10.1371/journal.pbio.1000412) for reporting work involving animal research. Where applicable, authors should refer to any relevant reporting standards documents in this form.

If you have any questions, please consult our Journal Policies and/or contact us: [editorial@elifesciences.org](mailto:editorial@elifesciences.org).

**Sample-size estimation**

- You should state whether an appropriate sample size was computed when the study was being designed
- You should state the statistical method of sample size computation and any required assumptions
- If no explicit power analysis was used, you should describe how you decided what sample (replicate) size (number) to use

Please outline where this information can be found within the submission (e.g., sections or figure legends), or explain why this information doesn’t apply to your submission:

For the *in vivo* drug screen, the number of larvae treated/condition was determined on a practical basis. We found that any more than 4 larvae/well in a 96-well plate increased the likelihood of larval death. Since the biosoter is only able to recover the larvae ~95% of the time, we decided that, to obtain information on at least 3 larvae, 4 larvae were required.

For other quantitative larvae-based experiments, we tried to adhere to approximately 50 larvae/treatment group. Again, this was mostly based on practicality. We wanted to observe enough larvae to capture the representative response to treatment while allowing for biological variation. For some experiments, like the glomerular filtration rate (GFR) assessment, fifty was the number of larvae that could realistically be injected within the experimental timeframe.

**Replicates**

- You should report how often each experiment was performed
- You should include a definition of biological versus technical replication
- The data obtained should be provided and sufficient information should be provided to indicate the number of independent biological and/or technical replicates
- If you encountered any outliers, you should describe how these were handled
- Criteria for exclusion/inclusion of data should be clearly stated
- High-throughput sequence data should be uploaded before submission, with a private link for reviewers provided (these are available from both GEO and ArrayExpress)

Please outline where this information can be found within the submission (e.g., sections or figure legends), or explain why this information doesn’t apply to your submission:

The number of times an experiment was performed, and the number of animals used per treatment group (where applicable) are reported in the corresponding figure legends.

Outliers were always reported as a part of the dataset.

**Statistical reporting**

- Statistical analysis methods should be described and justified
- Raw data should be presented in figures whenever informative to do so (typically when N per group is less than 10)
- For each experiment, you should identify the statistical tests used, exact values of N, definitions of center, methods of multiple test correction, and dispersion and precision measures (e.g., mean, median, SD, SEM, confidence intervals; and, for the major substantive results, a measure of effect size (e.g., Pearson's r, Cohen's d)
- Report exact p-values wherever possible alongside the summary statistics and 95% confidence intervals. These should be reported for all key questions and not only when the p-value is less than 0.05.

Please outline where this information can be found within the submission (e.g., sections or figure legends), or explain why this information doesn’t apply to your submission:

Statistical tests are reported in the corresponding figure legends.

Full statistical details are reported in supplementary table 2.

(For large datasets, or papers with a very large number of statistical tests, you may upload a single table file with tests, Ns, etc., with reference to sections in the manuscript.)

**Group allocation**

- Indicate how samples were allocated into experimental groups (in the case of clinical studies, please specify allocation to treatment method); if randomization was used, please also state if restricted randomization was applied
- Indicate if masking was used during group allocation, data collection and/or data analysis

Please outline where this information can be found within the submission (e.g., sections or figure legends), or explain why this information doesn’t apply to your submission:

Blinding was not used in this manuscript, as all testing was quantitative and all experiments were designed to minimize the impact of any bias. No randomization was used in this manuscript.

Treatment groups were created by aspirating larvae from pooled plates of larvae without microscopic observation. Due to the small size of the larvae, this was considered to be random.

**Additional data files (“source data”)**

- We encourage you to upload relevant additional data files, such as numerical data that are represented as a graph in a figure, or as a summary table
- Where provided, these should be in the most useful format, and they can be uploaded as “Source data” files linked to a main figure or table
- Include model definition files including the full list of parameters used
- Include code used for data analysis (e.g., R, MatLab)
- Avoid stating that data files are “available upon request”

Please indicate the figures or tables for which source data files have been provided:

The code used in R for the dose response curves found in Figure 1 and Supplementary Figure 1 can be found in the Materials and Methods section of the manuscript.
